# Comparative *in vitro* activity of various antibiotic against planktonic and biofilm and the gene expression profile in *Pseudomonas aeruginosa*

**DOI:** 10.3934/microbiol.2023017

**Published:** 2023-03-30

**Authors:** Mohammad Abu-Sini, Mohammad A. Al-Kafaween, Rania M. Al-Groom, Abu Bakar Mohd Hilmi

**Affiliations:** 1 Department of Pharmacy, Faculty of Pharmacy, Al-Zaytoonah University of Jordan, Amman, Jordan; 2 Department of Medical Laboratory Science, Faculty of Allied Medical Sciences, Zarqa University, Zarqa, Jordan; 3 Department of Allied Medical Sciences, Zarqa University College, Balqa Applied University, Al-Salt, Jordan; 4 Department of Biomedicine, Faculty of Health Sciences, Universiti Sultan Zainal Abidin, Terengganu, Malaysia

**Keywords:** *Pseudomonas aeruginosa*, biofilm, antibiotics, RT-qPCR, virulence genes

## Abstract

*P. aeruginosa* is an opportunistic pathogen that is commonly found in nosocomial infections. The purpose of this study was to investigate the effects of seven antibiotics on *P. aeruginosa* planktonic growth, biofilm formation, and the expression of virulence factors. These antibiotics included Ciprofloxacin (CP), Amikacin (AMK), Vancomycin (VAN), Tetracycline (TET), Gentamicin (GEN), Erythromycin (Ery), and Clindamycin (CLI). Antibiotic susceptibility testing, Minimum Bactericidal Concentration (MBC), Minimum Inhibitory Concentration (MIC), growth curve, time-kill curve, biofilm inhibition and reduction assay, and RT-qPCR were used to assess the effects of these antibiotics on *P. aeruginosa* planktonic and biofilm. The clear zones of inhibition against *P. aeruginosa* for the CP, AMK, VAN, TET, GEN, Ery, and CLI were 26 mm, 20 mm, 21 mm, 22 mm, 20 mm, 25 mm and 23 mm, respectively. The MIC values for CP, AMK, VAN, TET, GEN, Ery and CLI against *P. aeruginosa* ranged from 0.25 to 1 µg/mL while the MBC values ranged from 1 and 0.5 to 2 µg/mL respectively. The growth, total viable counts (TVCs), bacterial adhesion and biofilm formation of *P. aeruginosa* were reduced after exposure to all the tested antibiotics in a dose-dependent manner. The RT-qPCR analysis showed that all the tested antibiotics share a similar overall pattern of gene expression, with a trend toward reduced expression of the virulence genes of interest (*lasR, lasI, fleN, fleQ and fleR, oprB* and *oprC*) in *P. aeruginosa*. The results indicate that all of the tested antibiotics possess antimicrobial and anti-biofilm activities, and that they may be multiple inhibitors and moderators of *P. aeruginosa* virulence via a variety of molecular targets. This deduction requires to be investigated *in vivo*.

## 1. Introduction

*P. aeruginosa* is an opportunistic pathogen that may persist in a variety of environments, including hospitals. It is one of the most prevalent pathogens identified from nosocomial infections [1],[2]. The potential of *P. aeruginosa* to colonize medical equipment and human tissues while developing in resistant communities known as biofilms is a worldwide public health problem [3],[4]. In such an environmental niche, the bacterial communities are regulated by various biological processes and use advanced genotypic events to promote different molecular mechanisms and phenotypes that are necessary for survival in the new environment during pathogenesis and antibiotic treatment [5]. Thus, a biofilm is defined as a population of bacteria enclosed inside a self-secreted polymeric extracellular material matrix that is irrevocably adhered to a surface and difficult to remove with a gentle rinsing [6],[7].

Biofilm matrix formation and bacterial growth are influenced by variables such as nutrition availability and hydrodynamic circumstances. Cooperative interactions between species result in a variety of biofilm growth phases, structures, and functions [8],[9]. Because biofilms are polymicrobial, there is intense competition for resources and space [10]. The cohabitation of many bacteria on a surface increases cooperative behaviors such as metabolic cooperation, horizontal gene transfer, and other synergies, resulting in an improved ability of microorganisms to survive and fight antimicrobial chemicals [11],[12]. Resistance to antibiotics is approximately 1000 fold more in attached bacteria than planktonic cells because of an increase in mutation rates, upregulation of efflux pumps, decrease in metabolic activity, and other physical reasons [13]. The resistance mechanism is unique to biofilm-encapsulated bacteria as the biofilm phenotype provides a protective advantage [13],[14]. Bacteria alter gene expression during biofilm development adaption, encouraging phenotypically different behavior compared to planktonic counterparts.

Bacterial communication via the quorum sensing (QS) network is essential during biofilm formation, namely in controlling the genes involved in biofilm growth [15],[16]. According to the National Institutes of Health (NIH), bacterial biofilms are implicated with 65% of microbial diseases and more than 80% of chronic infections [17],[18]. The objective of this study was to (a) determine the antimicrobial activity of CP, AMK, VAN, TET, GEN, Ery against *P. aeruginosa* planktonic and biofilm stages and (b) estimate the impacts of these antibiotics on the expression of virulence genes in *P. aeruginosa*

## 2. Materials and methods

### 2.1. Bacterial strains and culture conditions

A reference strain of *Pseudomonas aeruginosa* (ATCC 9027) was purchased from the American Type Culture Collection (ATCC). The strain was streaked on Luria-Bertani (LB) agar and incubated for 24 hrs at 37 °C. After incubation, the strain was inoculated into sterile LB broth and incubated at 37 °C for 12 hrs. The bacterial suspension was then adjusted to be 0.5 McFarland. Then, the strain was stored in Luria-Bertani (LB) broth containing 20% (v/v) glycerol at −80 °C [19]–[21].

### 2.2. Antibiotic susceptibility testing

The antibiotic-susceptibility testing was performed using Muller Hinton agar (MHA) by the modified Kirby-Bauer disc diffusion method following Clinical and Laboratory Standard guidelines (CLSI) [22],[23]. The antibiotics tested in this study were ciprofloxacin (5 µg), gentamicin (10 µg), tetracycline (30 µg), amikacin (30 µg), clindamycin (10 µg), erythromycin (15 µg), and vancomycin (30 µg). A suspension of *P*. *aeruginosa* adjusted to 0.5 McFarland standards and streaked on Mueller Hinton Agar (MHA) plates using sterile swabs and antibiotic discs were placed on top. The plates were incubated for 24 hrs at 37 °C. Distilled water was used as a negative control. The diameter of the zone of inhibition for each antibiotic was measured by digital venire caliper. The experiment was carried out in triplicate [20],[22],[23].

### 2.3. MIC and MBC determination

Different concentrations (8, 4, 2, 1, 0.5, 0.25 and 0.125 µg/mL) for CP, AMK, VAN, TET, GEN, Ery and CLI were prepared. A suspension of *P*. *aeruginosa* was adjusted to be 0.5 McFarland standards as described previously. Briefly, 100 µL of each antibiotic dispensed with 100 µL of suspension into microtiter plate. Bacteria with antibiotic was used as positive control and antibiotic with broth was served as negative control. Then, the plates were incubated for 24 hrs at 37 °C. After incubation time was done, microplate reader was used to measure the optical density (OD) at 540 nm wavelength. The MIC value was set as the minimum concentration of the antimicrobial necessary to prevent bacterial growth after 24 hrs of incubation at 37 °C. For MBC determination, samples in the wells without turbidity were spread onto LB agar and incubated at 37 °C for 24 hrs. The minimum concentration that resulted in no *P*. *aeruginosa* growth on the plate was defined as MBC. The experiment was performed in triplicate [21],[24]–[27].

### 2.4. Growth curve determination

Briefly, P. aeruginosa suspension treated with CP, GEN, TET, AMK, CLI, Ery and VAN (0, 1/4 × MIC, 1/2 × MIC and 1 × MIC) and incubated statically at 37 °C in a 96-well plate for 24 hrs. The optical density at 540 nm (OD_540_ nm) of each sample was measured at 2-h intervals using a microplate reader. The concentrations that exerted no significant effects on *P. aeruginosa* growth were considered as the sub-inhibitory concentrations (SICs) of CP, GEN, TET, AMK, CLI, Ery and VAN [20],[23],[28],[29].

### 2.5. Time-kill curve

The time-kill assay was performed as described by Shi et al., (2016) with few modifications. Microcentrifuge tubes containing control, MIC, and MBC values of CP, AMK, VAN, TET, GEN, Ery and CLI were incubated with *P. aeruginosa* suspension (1 x 10^6^ cfu/mL). Aliquots from above mixture were taken at a different time interval (0, 3, 6, 9, 12, 15, and 18, 21, 24 h), serially diluted and plated on the LB agar plate. Following 24 hrs of incubation, colony counting was performed by using colony counter. Time-kill curve was plotted by assessing the log_10_ CFU (colony forming units) versus time [20],[21],[23],[28],[30].

### 2.6. Biofilm inhibition-crystal violet assay

*P. aeruginosa* were grown for 48 hrs in LB broth. A mixture of bacterial broth and 8, 4, 2, 1, 0.5, 0.25 and 0.125 µg/mL concentration of CP, AMK, VAN, TET, GEN, Ery and CLI was added to the 96-well plates. Incubation was done overnight at 37 °C. After 12 hrs incubation, culture supernatant was removed and washed with PBS to remove unbound bacteria. The bound bacteria were fixed with methanol for 10 minutes and air dried. Bound bacteria were stained using crystal violet (0.1% w/v) for 3 minutes, and unbound dye was washed away with distilled water. The plate was air-dried, and bound dye was dissolved in 95% (v/v) ethanol. The optical density (OD) was read at 540 nm using a microplate reader. The percentage of biofilm inhibition was calculated by following formulas as mentioned below. The experiment was repeated in triplicate [20],[21],[23],[31],[32].

$$\text{Percentage inhibition}\left(\%\right)=\frac{\text{OD }\left(\text{positive control value}\right)-\text{OD }\left(\text{sample value}\right)\times \text{1}00\%}{\text{OD }\left(\text{positive control value}\right)}$$ (1)

### 2.7. Biofilm reduction-crystal violet assay

Briefly, overnight grown *P. aeruginosa* having a concentration of 0.5 McFarland standards was inoculated in LB broth in each well and 200 µL of sterile distilled water was added into wells to reduce the water loss. The plate was then kept at 37 °C for 72 hrs to allow the bacteria to grow and form the biofilms the well's bottom surface. Each well was later replaced with 8, 4, 2, 1, 0.5, 0.25 and 0.125 µg/mL concentrations of CP, AMK, VAN, TET, GEN, Ery and CLI in culture medium and culture medium alone (control). The incubation was continued for another 24 hrs under the same condition. After 24 hrs, the supernatant was discarded, and wells were rinsed with PBS. Fixation was done with ethanol as described previously. The percentage of biofilm reduction was calculated by following formulas as mentioned below [21],[24],[28],[33]–[35].

$$\text{Percentage reduction}\left(\%\right)=\frac{\text{OD }\left(\text{positive control value}\right)-\text{OD }\left(\text{sample value}\right)\times \text{1}00\%}{\text{OD }\left(\text{positive control value}\right)}$$ (2)

### 2.8. Extraction of RNA for RT-qPCR

*P. aeruginosa* was grown in duplicate in 5 mL LB broth with MIC and MBC of CP, AMK, VAN, TET, GEN, Ery and CLI for 24 hrs at 37 °C. After incubation, the samples were re-suspended in 500 mL PBS and vortexed for 2 minutes to break up cell aggregates. Then, the RNA was extracted using the SV total RNA extraction kit (Promega, UK). The bacterial total RNA integrity was checked by NanoDrop, and each RNA sample was adjusted to give a final concentration of 100 ng/µL. The primers were used for *P. aeruginosa* as shown in Table 1. Reverse RNA transcription was performed with Oligo (dT)_15_ primers and Random Primers. Total RNA samples were converted to cDNA using a high capacity RNA to cDNA conversion kit (Promega, UK) and quantitative PCR expression analysis as following the manufacturer's instructions (Promega, UK). Densitometry was performed using the Applied Biosystems StepOne Software v2.3 to determine the level of relative gene expression in *P. aeruginosa* samples. A modified 2^−ΔΔ^ Ct method was used. All reactions were carried out in triplicate, and the genes' expressions were analyzed with reference to the housekeeping gene expression [21],[36]–[45].

**Table 1.** Primers for RT-qPCR of *P. aeruginosa*

Gene name	Amplicon Size (bp)	Annealing temp (°C)	Direction	Primer sequence (5′→ 3′)
*oprB*	140	54	Forward	TGACGACGACAAGACAGGAC
			Reverse	GGTCGTTGGAAAGGTTCTTG
*oprC*	105	55	Forward	GCCTGAACATCCTCACCAAC
			Reverse	CGGTGAGCTTGTCGTAGGTT
*fleN*	137	56	Forward	GAGCCGTATACGAGGCATTC
			Reverse	GTGTTGGACCAGTCGTTCG
*fleQ*	134	54	Forward	AAGGACTACCTGGCCAACCT
			Reverse	CCGTACTTGCGCATCTTCTC
*fleR*	109	55	Forward	ACAGCCGCAAGATGAACCT
			Reverse	TGGATGGCGTTGTCGAGTT
*lasR*	129	54	Forward	CGGTTTTCTTGAGCTGGAAC
			Reverse	TCGTAGTCCTGGCTGTCCTT
*lasI*	129	54	Forward	ATGATCGTACAAATTGGTCGGC
			Reverse	GTCATGAAACCGCCAGTCG
*rpoD* (Reference gene)	146	53	Forward	GCGACGGTATTCGAACTTGT
			Reverse	CGAAGAAGGAAATGGTCGAG

### 2.9. Statistical analysis

Data were presented as mean ± standard deviation. To compare the treatment and control groups, an independent student t-test from SPSS version 20 was employed. The significance level was set at *P* < 0.05.

## 3. Results

### 3.1. Antibiotic susceptibility testing

As shown in Table 2, the antibiotic susceptibility of CP, AMK, VAN, TET, GEN, Ery and CLI were showed varying degrees of inhibitory activity against *P. aeruginosa*. The inhibition zone of CP, AMK, VAN, TET, GEN, Ery and CLI against the *P. aeruginosa* were 26 mm, 20 mm, 21 mm, 22 mm, 20 mm, 25 mm and 23 mm respectively. The most antibiotic effective on *P. aeruginosa* was Ciprofloxacin (CP).

**Table 2.** Zones of inhibition dimeter (mm) of the tested antibiotics against *P. aeruginosa*.

Antibiotics	1st	2nd	3rd	Main value
Ciprofloxacin (CP)	26 mm ± 1.1	26 mm ± 1.0	26 mm ± 1.2	26 mm ± 1.1
Amikacin (AMK)	20 mm ± 0.3	20 mm ± 0.3	20 mm ± 0.3	20 mm ± 0.3
Vancomycin (VAN)	21 mm ± 0.6	21 mm ± 0.6	21 mm ± 0.5	21 mm ± 0.6
Tetracycline (TET)	22 mm ± 0.7	22 mm ± 0.8	22 mm ± 0.7	22 mm ± 0.7
Gentamicin (GEN)	20 mm ± 0.6	20 mm ± 0.5	20 mm ± 0.6	20 mm ± 0.6
Erythromycin (Ery)	25mm ± 0.9	25 mm ± 0.9	25 mm ± 0.8	25 mm ± 0.9
Clindamycin (CLI)	23 mm ± 0.8	23 mm ± 0.8	23 mm ± 0.8	23 mm ± 0.8

Ciprofloxacin (CP), Amikacin (AMK), Vancomycin (VAN), Tetracycline (TET), Gentamicin (GEN), Erythromycin (Ery) and Clindamycin (CLI), Mean ±standard deviation (SD), n =3

### 3.2. MIC and MBC determination

The antimicrobial activity of CP, AMK, VAN, TET, GEN, Ery and CLI against *P. aeruginosa* were performed using the standard broth dilution method. The MIC value of CP and AMK were 0.25 µg/mL, and 0.5 µg/mL for VAN and TET, and 1.0 µg/mL for GEN, Ery and CLI against *P. aeruginosa*. The MBC value of CP and AMK was 0.5 µg/mL, and 1.0 µg/mL for VAN and TET, and 2.0 µg/mL for GEN, Ery and CLI against *P. aeruginosa* (Table 3).

**Table 3.** MIC and MBC values of antibiotics against *P. aeruginosa*.

MBC (µg/mL)	MIC (µg/mL)	Antibiotics
0.5	0.25	CP
0.5	0.25	AMK
1.0	0.5	VAN
1.0	0.5	TET
2.0	1.0	GEN
2.0	1.0	Ery
2.0	1.0	CLI

Ciprofloxacin (CP), Amikacin (AMK), Vancomycin (VAN), Tetracycline (TET), Gentamicin (GEN), Erythromycin (Ery) and Clindamycin (CLI). Mean ± standard deviation (SD), n = 3

### 3.3. Growth curve determination

**Figure 1.**
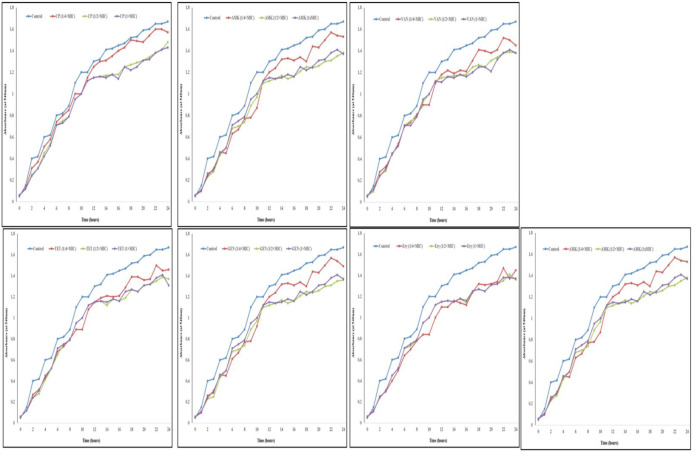
Growth curves of *P. aeruginosa* in the presence of antibiotics at the concentrations of 0 (control), 1/4 × MIC, 1/2 × MIC and 1 × MIC for 24 hrs. Ciprofloxacin (CP), Amikacin (AMK), Vancomycin (VAN), Tetracycline (TET), Gentamicin (GEN), Erythromycin (Ery) and Clindamycin (CLI). Mean ±standard deviation (SD), n = 3

Effects of CP, AMK, VAN, TET, GEN, Ery and CLI (0, 1/4 × MIC, 1/2 × MIC and 1 × MIC) on the growth of *P. aeruginosa* were evaluated by growth curve assay. As shown in Figure 1, after *P. aeruginosa* exposure to all the antibiotics at the concentration of 1/4 × MIC, 1/2 × MIC and 1 × MIC, the optical density (OD) value of untreated samples was increased. *P. aeruginosa* growth curves in the presence of all the antibiotics (1/4 × MIC) were similar to that of the control, indicating that all the antibiotics (1/4 × MIC) showed no significant effect on *P. aeruginosa* growth. In the presence of 1/2 × MIC and 1 × MIC of CP, AMK, VAN, TET, GEN, Ery and CLI, the OD value was almost unchanged in 6 hrs and decreased after 6 hrs, suggesting that all the antibiotics at 1/2 × MIC and 1 × MIC could inhibit the growth of *P. aeruginosa*. As a result, these concentrations were considered as SICs of antibiotics against *P. aeruginosa*.

### 3.4. Time-kill curve

**Figure 2.**
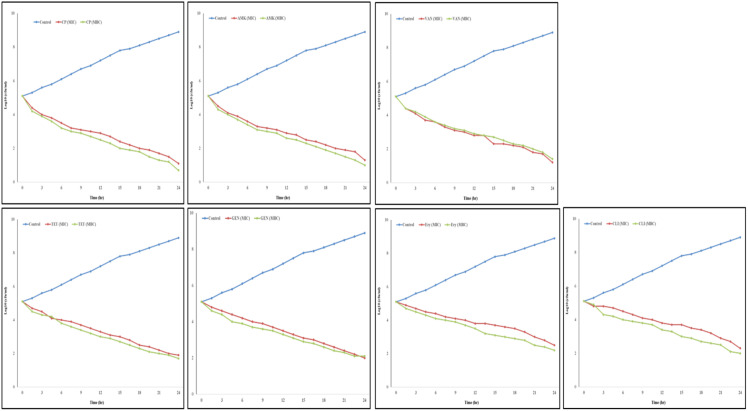
Time-kill curves of antibiotics against *P. aeruginosa*. Samples were treated with antibiotics at the concentrations of 0 (control), 1 × MIC and 2 × MIC for 24 hrs. Ciprofloxacin (CP), Amikacin (AMK), Vancomycin (VAN), Tetracycline (TET), Gentamicin (GEN), Erythromycin (Ery) and Clindamycin (CLI), Mean ±standard deviation (SD), n = 3

Time-kill test the time-kill assay was used to assess the antibacterial activity of CP, AMK, VAN, TET, GEN, Ery, and CLI against *P. aeruginosa*. This test was carried out after the bacteria were exposed to MIC and MBC concentrations of all antibiotics for different time intervals. At the MIC concentration, bacterial growth was inhibited in a time-dependent manner, but at the MBC concentration, 99% inhibition was achieved within 24 hrs. As shown in Figure 2, the starting concentration of *P. aeruginosa* in all samples was 5.1-log cfu/mL. Without antibiotics, the number of bacterial cells grew to 8.9-log cfu/mL after 24 hrs. However, treatment with CP, AMK, VAN, TET, GEN, Ery, and CLI (MIC) resulted in a significant decrease in the growth of *P. aeruginosa*. After 24 hrs of treatment with CP, AMK, VAN, TET, GEN, Ery, and CLI (MIC), the bacterial count was decreased to 2.3-log cfu/mL. In addition, after exposure to CP, AMK, VAN, TET, GEN, Ery and CLI at MBC, the bacterial number decreased to 2.0-log cfu/mL.

### 3.5. Biofilm inhibition-crystal violet assay

**Figure 3.**
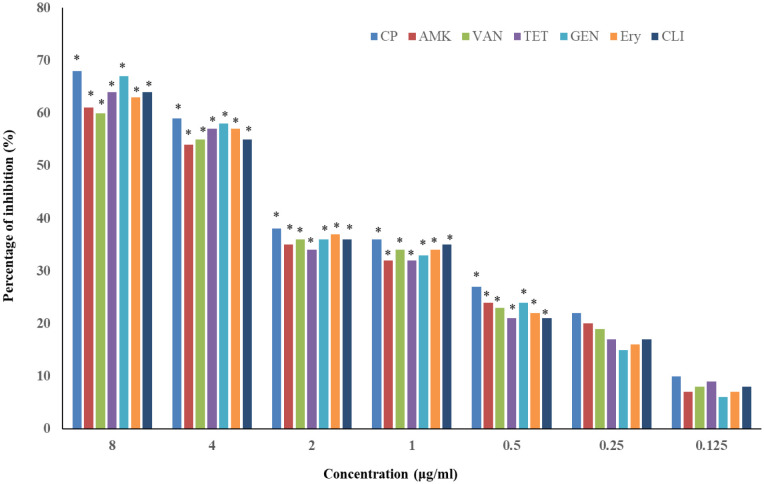
Inhibition of biofilm formation in *P. aeruginosa* in the presence of antibiotics. Ciprofloxacin (CP), Amikacin (AMK), Vancomycin (VAN), Tetracycline (TET), Gentamicin (GEN), Erythromycin (Ery) and Clindamycin (CLI), Mean ± standard deviation (SD), n = 3. Asterisks; *P < 0.05 indicate statistically significant difference between treated and control samples.

The biofilm reduction assay showed that 8, 4, 2, 1 and 0.5 µg/mL concentration of CP, AMK, VAN, TET, GEN, Ery, and CLI significantly reduced the number of attached *P. aeruginosa* cells, up to 60% (P < 0.05) relative to the control group. Furthermore, *P. aeruginosa* biofilm development was not significantly reduced following treatment with 0.5 and 0.25 µg/mL of CP, AMK, VAN, TET, GEN, Ery, and CLI. The lowest dose of CP, AMK, VAN, TET, GEN, Ery, and CLI that inhibited *P. aeruginosa* biofilm was determined to be 0.125 µg/mL (Figure 3).

### 3.6. Biofilm reduction-crystal violet assay

**Figure 4.**
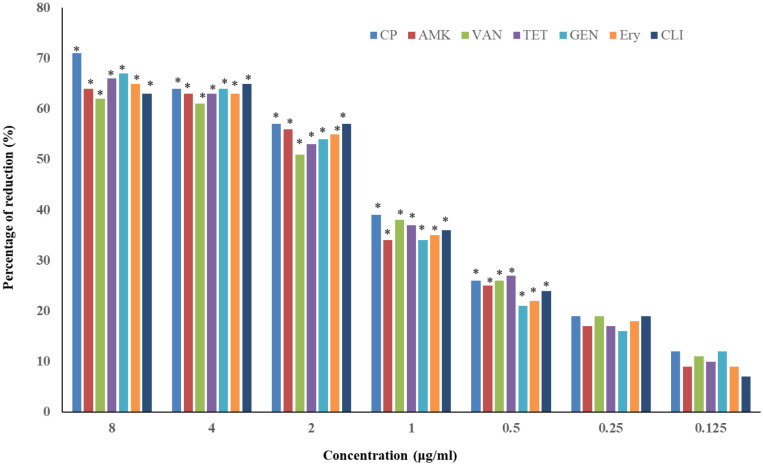
Reduction of *P. aeruginosa* biofilm after exposure to antibiotics. Ciprofloxacin (CP), Amikacin (AMK), Vancomycin (VAN), Tetracycline (TET), Gentamicin (GEN), Erythromycin (Ery) and Clindamycin (CLI), Mean ± standard deviation (SD), n = 3. Asterisks; *P < 0.05 indicate statistically significant difference between treated and control samples.

As shown in Figure 4, at 8, 4, 2, 1 and 0.5 µg/mL concentration of CP, AMK, VAN, TET, GEN, Ery, and CLI significantly reduced (P < 0.05) the adhesion of *P. aeruginosa* biofilm compared to the control. However, 0.25 and 0.125 µg/mL concentration of CP, AMK, VAN, TET, GEN, Ery, and CLI did not show any significant effect.

### 3.7. Gene expression profile

**Figure 5.**
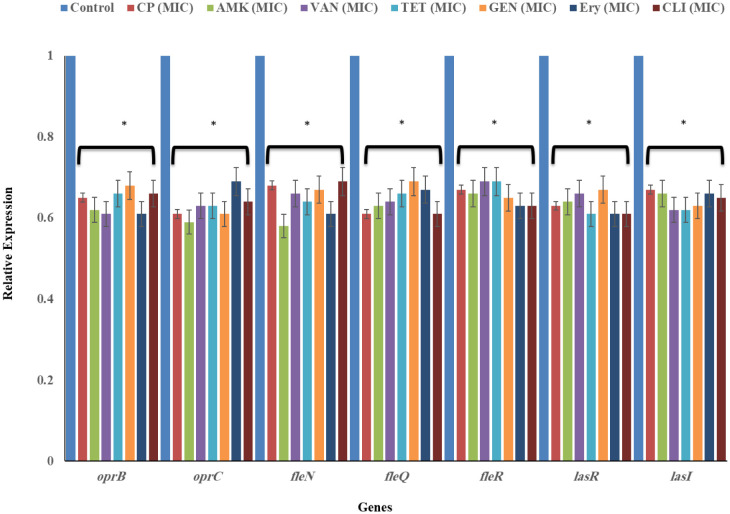
The level of gene expression in *P. aeruginosa* in the absence and presence of MIC of all the tested antibiotics, as assessed by RT-qPCR. Ciprofloxacin (CP), Amikacin (AMK), Vancomycin (VAN), Tetracycline (TET), Gentamicin (GEN), Erythromycin (Ery) and Clindamycin (CLI). Data is mean ± SD of these independent experiments. Significant difference indicated as *P < 0.05, **P < 0.01 between control versus treated samples.

**Figure 6.**
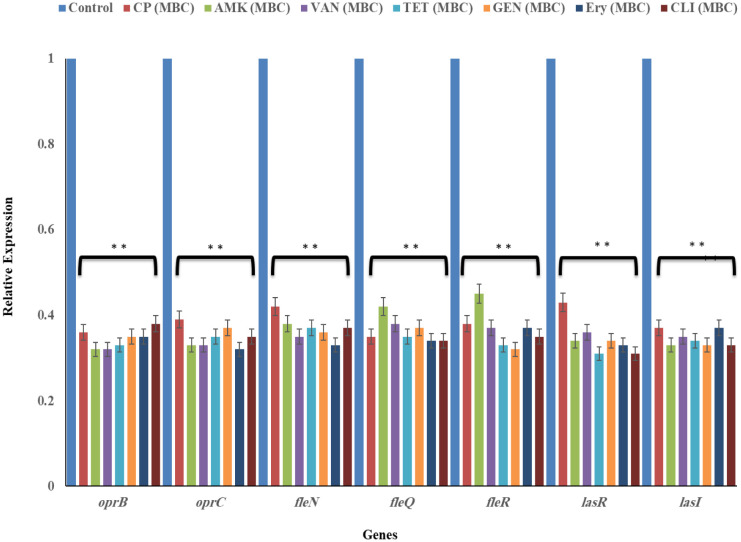
The level of gene expression in *P. aeruginosa* in the absence and presence of MBC of all the tested antibiotics, as assessed by RT-qPCR. Ciprofloxacin (CP), Amikacin (AMK), Vancomycin (VAN), Tetracycline (TET), Gentamicin (GEN), Erythromycin (Ery) and Clindamycin (CLI). Data is mean ± SD of three independent experiments. Significant difference indicated as *P < 0.05, **P < 0.01 between control versus treated samples.

RT-qPCR was used to study the influence of antibiotics (at MIC and MBC) on virulence factor expression levels of the microcolony formation, motility, outer membrane protein and biofilm involved genes in *P. aeruginosa*. All the tested antibiotics (CP, AMK, VAN, TET, GEN, Ery, and CLI) caused a reduction in the gene expression of all virulence factors in dose-dependent manner. The antibiotics significantly reduced *oprB, oprC, fleN, fleQ, fleR, lasR* and *lasI* expression at MICs (*P* < 0.05) and MBCs (*P* < 0.01) concentration (Figures 5 and 6).

#### 3.7.1. Genes involved in biofilm formation were suppressed after exposure to antibiotics

The inability of *P. aeruginosa* to form biofilm in respond to MICs and MBCs TH treatment was demonstrated by two biofilm-forming genes, *lasR* and *lasI*. The *lasR* and *lasI* were significantly (*P* < 0.05) downregulated in *P. aeruginosa* after being treated with MICs (*P* < 0.05) and MBCs (*P* < 0.01) concentration of all the tested antibiotics (Figures 5 and 6).

#### 3.7.2. Flagella-associated genes were suppressed by antibiotics

Three investigated flagella genes: *fleN, fleQ* and *fleR* of *P. aeruginosa* demonstrated the significant reduction of gene expressions in response to exposure to MICs (*P* < 0.05) and MBCs (*P* < 0.01) concentration of all the tested antibiotics (Figures 5 and 6).

#### 3.7.3. Genes associated with outer-membrane protein were suppressed following treatment with antibiotics

Two investigated genes; *oprB* and *oprC* associated with the outer membrane protein (cell wall stability, diffusion and virulence) of *P. aeruginosa* showed the significant reduction (*P* < 0.05) of gene expressions following treatment with MICs (*P* < 0.05) and MBCs (*P* < 0.01) concentration of all the tested antibiotics (Figures 5 and 6).

## 4. Discussion

*P. aeruginosa* is an important human opportunistic pathogen that causes acute and persistent infections, particularly in immunocompromised patients. Overuse of antibiotics in recent decades has resulted in the evolution of resistant forms of these bacteria [46]. Biofilm development is one of these bacteria's strategies for reducing the effect of antibiotic treatment. *P. aeruginosa* biofilm is a structure made up of several biomolecules that develop as they come together. Bacterial attachment factors, extracellular polysaccharides (EPS), and extracellular DNA (eDNA) all play a role in the creation of this structure [47]. Two-component regulatory systems and bacterial quorum sensing regulate the expression of key genes during the secretion and development of biofilm components. Failure in these processes results in a stop in biofilm formation or a faulty structure [48].

The MIC and MBC values indicated that CP, AMK, VAN, TET, GEN, Ery, and CLI demonstrated effective antimicrobial activity against *P. aeruginosa*. In the present study, CP, AMK, VAN, TET, GEN, Ery, and CLI were demonstrated to show antimicrobial activity against *P. aeruginosa* with the MIC 0.25, 0.25, 0.5, 0.5, 1.0, 1.0 and 1.0 µg/mL respectively and with the MBC 0.5, 0.5, 1.0, 1.0, 2.0, 2.0 and 2.0 µg/mL respectively. Study by [49] showed that the MIC and MBC values of Ciprofloxacin were 0.5 µg/mL and 8 µg/mL against *P. aeruginosa* [49]. Another study explored that the MIC value of Ciprofloxacin and Azithromycin was between 2 to >64 µg/mL and the MIC value of Erythromycin was between 8 to >64 µg and the MBC value was between 2 to >64 µg/mL against *P. aeruginosa* [50].

Growth curves with (1/2 MIC and 1 MIC) of all tested antibiotics resulted in a lower growth rate and total cell number of *P. aeruginosa* during a 24 hrs, compared to cells grown without antibiotics. The time-kill assay showed that CP, AMK, VAN, TET, GEN, Ery, and CLI have bactericidal effects against the strain of *P. aeruginosa* at MBC concentration. Bacterial inhibition increases with antibiotic concentration and incubation time. A biofilm is an aggregation of one or more species of microorganisms adhered to a surface, as compared to planktonic bacteria, which exist as individual organisms [20],[51]. Biofilm-forming bacteria are reported to be 100–1000 times more resistant to antibiotics than planktonic bacteria [13],[14]. In this study, biofilm inhibition and reduction-crystal violet assay suggested that all the tested antibiotics at 8, 4, 2, 1 and 0.5 µg/mL concentrations were significantly able to decrease *P. aeruginosa* biofilm formation. Studies evaluating the efficiency of antibiotic mono or combination treatment against biofilm infections are important and valued by the medical community. However, only a few studies have performed such research in models mimicking real biofilm infections. Aminoglycoside in combination with b-lactam antibiotics is often used intravenously in hospitals as the major treatments against *P. aeruginosa* infection. Furthermore, biofilm bacteria, but not adherent bacteria, were significantly more resistant to two- or three antibiotic combination treatments than the planktonic bacteria [52], [53].

In general, concentration-dependent killing was demonstrated in our study. It was possible to remove more than 50% of the bacteria in mature *in vitro* biofilms after treated with 8, 4, 2, and 1 µg/mL concentration of antibiotic. Antibiotics were able to reduce the number of live bacteria at 8, 4, 2, 1 and 0.5 µg/mL concentrations suggesting that it has bactericidal. Effect of antibiotics (CP, AMK, VAN, TET, GEN, Ery, and CLI) on relative expression of genes (*oprB, oprC, fleN, fleQ, fleR, lasR* and *lasI*) associated with biofilm formation, motility, and outer membrane protein of *P. aeruginosa* was further investigated by RT-qPCR, and the result showed that transcriptional levels of seven related genes were significantly downregulated by antibiotics at MIC and MBC. These findings indicated that each antibiotic had the potential to be an anti-biofilm agent. At present, studies concerning anti-biofilm effect on *P. aeruginosa* by antibiotics are relatively limited, and therefore, more related investigations are required. In summary, antibiotics have good antibacterial and anti-biofilm activity against *P. aeruginosa*. antibiotics could inactivate *P. aeruginosa* cells. Furthermore, antibiotics at MIC and MBC could depress biofilm formation by *P. aeruginosa* and downregulate the transcriptional levels of related genes. This study indicated that antibiotics is an effective to control the contamination and infection caused by *P. aeruginosa*. Further studies focused on the anti-biofilm mechanism of antibiotics should be conducted.

## 5. Conclusion

Susceptibility testing of planktonic bacteria can be an impediment to the effective treatment of chronic caused by biofilm-forming pathogens. The clear zones of inhibition against *P. aeruginosa* for the CP, AMK, VAN, TET, GEN, Ery, and CLI were 26 mm, 20 mm, 21 mm, 22 mm, 20 mm, 25 mm and 23 mm, respectively. In addition, the MIC values for CP, AMK, VAN, TET, GEN, Ery and CLI against *P. aeruginosa* ranged from 0.25 to 1 µg/mL while the MBC values ranged from 1 and 0.5 to 2 µg/mL respectively. In this study, there is a minor variation in MIC and MBC between the antibiotics against *P. aeruginosa* and each antibiotic inhibited *P. aeruginosa* biofilm. In the current study, RT-qPCR analysis showed that all the tested antibiotics share a similar overall pattern of gene expression, with a trend toward reduced expression of the virulence genes of interest in *P. aeruginosa*. The findings suggest that these antibiotics may be potential anti-biofilm and anti-virulence agents for the treatment and regulation of *P. aeruginosa* infections. The current study findings might be validated using a combination of real-time PCR and microarray analysis. It would also be interesting to investigate the genes involved in biofilm formation, quorum sensing, and auto-inducers in *P. aeruginosa* and to find other gene expression pathways. However, further studies are warranted to study the effect of antibiotics on more virulent strains of *P. aeruginosa* and study it's in vivo efficacy in suitable animal models.
